# BCR activated CLL B cells use both CR3 (CD11b/CD18) and CR4 (CD11c/CD18) for adhesion while CR4 has a dominant role in migration towards SDF-1

**DOI:** 10.1371/journal.pone.0254853

**Published:** 2021-07-20

**Authors:** Zsuzsa Nagy-Baló, Richárd Kiss, Judit Demeter, Csaba Bödör, Zsuzsa Bajtay, Anna Erdei

**Affiliations:** 1 Department of Immunology, Eötvös Loránd University, Budapest, Hungary; 2 MTA-ELTE Immunology Research Group, Eötvös Loránd University, Budapest, Hungary; 3 MTA-SE Momentum Molecular Oncohematology Research Group, First Department of Pathology and Experimental Cancer Research, Semmelweis University, Budapest, Hungary; 4 Department of Internal Medicine and Oncology, Semmelweis University, Budapest, Hungary; European Institute of Oncology, ITALY

## Abstract

Chronic lymphocytic leukaemia (CLL) is the most common leukaemia in the western world. In previous studies, various proportion of patients was found to carry CD11b^+^ or CD11c^+^ B cells whose presence was an unfavourable prognostic factor. The exact mechanism however, how these receptors contribute to the pathogenesis of CLL has not been revealed so far. Here we analysed the role of CD11b and CD11c on B cells of CLL patients in the adhesion to fibrinogen and in the migration towards stromal cell derived factor-1 (SDF-1) and studied the role of CR4 in the adherence of the CD11c^+^ B cell line BJAB. We observed that both CR3 and CR4 mediate adhesion of the malignant B cells. Moreover, we found, that CR4 was strongly involved in the migration of the leukemic cells towards the chemoattractant SDF-1. Our data suggest that CR3 and CR4 are not only passive markers on CLL B cells, but they might contribute to the progression of the disease. Since the role of SDF-1 is prominent in the migration of CLL cells into the bone marrow where their survival is supported, our findings help to understand how the presence of CD11c on leukemic B cells can worsen the prognosis of chronic lymphocytic leukaemia.

## 1. Introduction

The role of complement receptors CR3 (CD11b/CD18, also known as Mac-1, α_M_β_2_) and CR4 (CD11c/CD18, also designated as p150,95; α_X_β_2_) are known to be involved in actin linked functions such as phagocytosis, adhesion or migration. These two receptors of the β_2_-integrin family have earlier been suggested to mediate overlapping functions, however, we propose that they rather should be considered as “non-identical twins” [1], because of the functional segregation between them. Namely, studying human monocytes, macrophages and dendritic cells we have found that the role of CR3 is dominant in the process of phagocytosis [2], while in adhesion to fibrinogen CR4 prevails over CR3 [3].

While their role is well documented in myeloid cell types, detailed studies aiming to reveal the expression and role of CR3 and CR4 on B lymphocytes have begun only recently. In the case of healthy donors it has been found, that these receptors are scarcely expressed on non-activated B lymphocytes [4, 5], but after activation by different stimuli they appear on certain B cell populations [5–10]. We found lately that activated human memory B lymphocytes use CR4 for adhesion, migration, and proliferation [11].

Moreover, these complement receptors have been detected in various B cell malignancies such as in Hodgkin’s lymphoma [12], hairy cell leukaemia [13] or chronic lymphocytic leukaemia (CLL) [4, 13–19]. Out of these CR3 and CR4 bearing B cell malignancies, CLL is the most frequent form of leukaemia in the western world with an age-adjusted incidence of 4.1/100 000. Heterogeneous clinical course is a hallmark of CLL, the majority of patients follow an indolent clinical course, while a proportion of patients experience rapid disease progression [20]. The disease is characterized by the accumulation of clonal memory B cells with a distinct immunophenotype (i.e. CD5, CD19, CD20, CD23) within the blood, lymph nodes, spleen, bone marrow and other lymphatic tissues [21, 22].‬

It has also been described that the expression of CR3 and CR4 on CLL B cells varies widely among patients and the level of the expression correlates with the progression of the disease [14–17]. It is assumed that the presence of these β_2_-integrins might contribute to the elevated adhesive and migratory capacity of the malignant cells, but the exact function of these receptors on CLL B cells is unexplained so far. Recently, our group demonstrated that both CR3 and CR4 are involved in the spreading of CpG-activated CLL B cells on fibrinogen [4]. Moreover, since activated B cells of healthy donors use CR4 for adhesion, migration, and proliferation [11], we suggest that CR4 may have a similar role in CLL B cells too, as malignant cells represent the activated state of their normal counterpart.

The aim of the present study was to investigate the expression and role of CR3 and CR4 on B lymphocytes of chronic lymphocytic leukaemia patients. Here we demonstrate that both CR3 and CR4 contribute to the adhesion of CLL B cells. Regarding migration towards the chemoattractant SDF-1 however, CR4 has a dominant role.

## 2. Results

### 2.1. Expression and function of CR3 and CR4 on the B cell line BJAB

**Fig 1:**
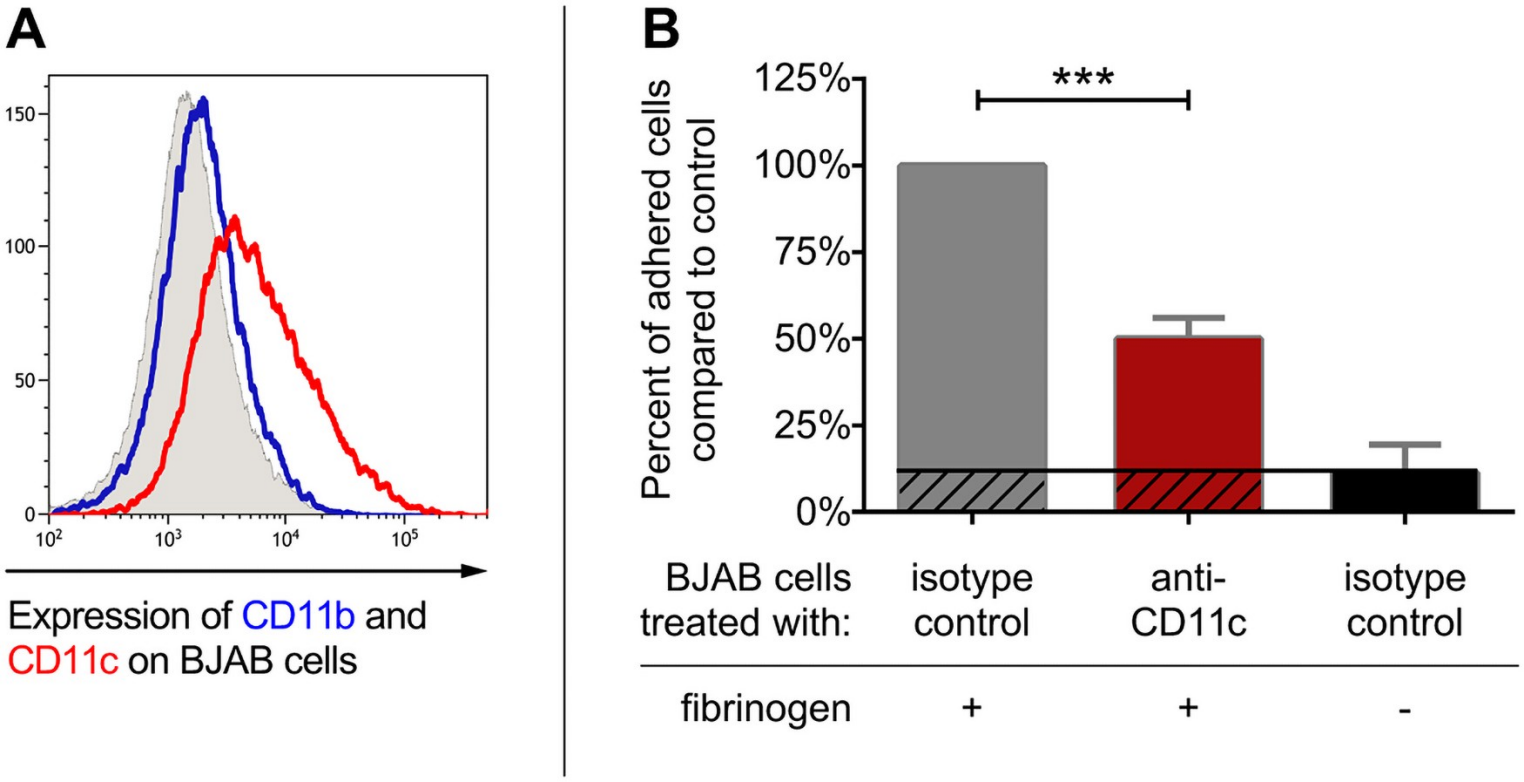
CR4 is expressed by the cell line BJAB and contributes to adherence. (A) CR3 (blue) and CR4 (red) expression was measured by flow cytometry. Result of one representative experiment of 5 independent one is shown. (B) BJAB cells were let to adhere to fibrinogen coated and PLL-PEG blocked surface in the presence of CD11c specific (red) or isotype control antibodies (grey). Results of 5 independent experiment are shown (mean+/− SD). One-way ANOVA with Tukey’s post-test was used to assess significant differences compared to control, * = p < 0.05; ** = p < 0.01; *** = p < 0.001.

Since CD11b and CD11c are known to be expressed by various malignant B cells [4, 12–19], first we tested whether the cells of the EBV-negative Burkitt-like lymphoma line BJAB could serve as a model for examining the role of CR3 or CR4 expressed by malignant human B cells. Flow cytometry measurements revealed that BJAB cells are positive for CD11c, but negative for CD11b (Fig 1A), similarly to the activated B cells of healthy donors [11]. As CR4 was found to be involved in the adhesion of activated normal B cells [11], we set out to measure the adhesive capacity of BJAB cells to fibrinogen, the natural ligand of CR4. We found that blocking CR4 by a CD11c specific antibody significantly decreased the number of adherent cells compared to control (Fig 1B). Thus, CR4 is expressed in a functionally active state on the Burkitt lymphoma-derived BJAB cells.

### 2.2. Expression of CR3 and CR4 on B cells of CLL patients

**Fig 2:**
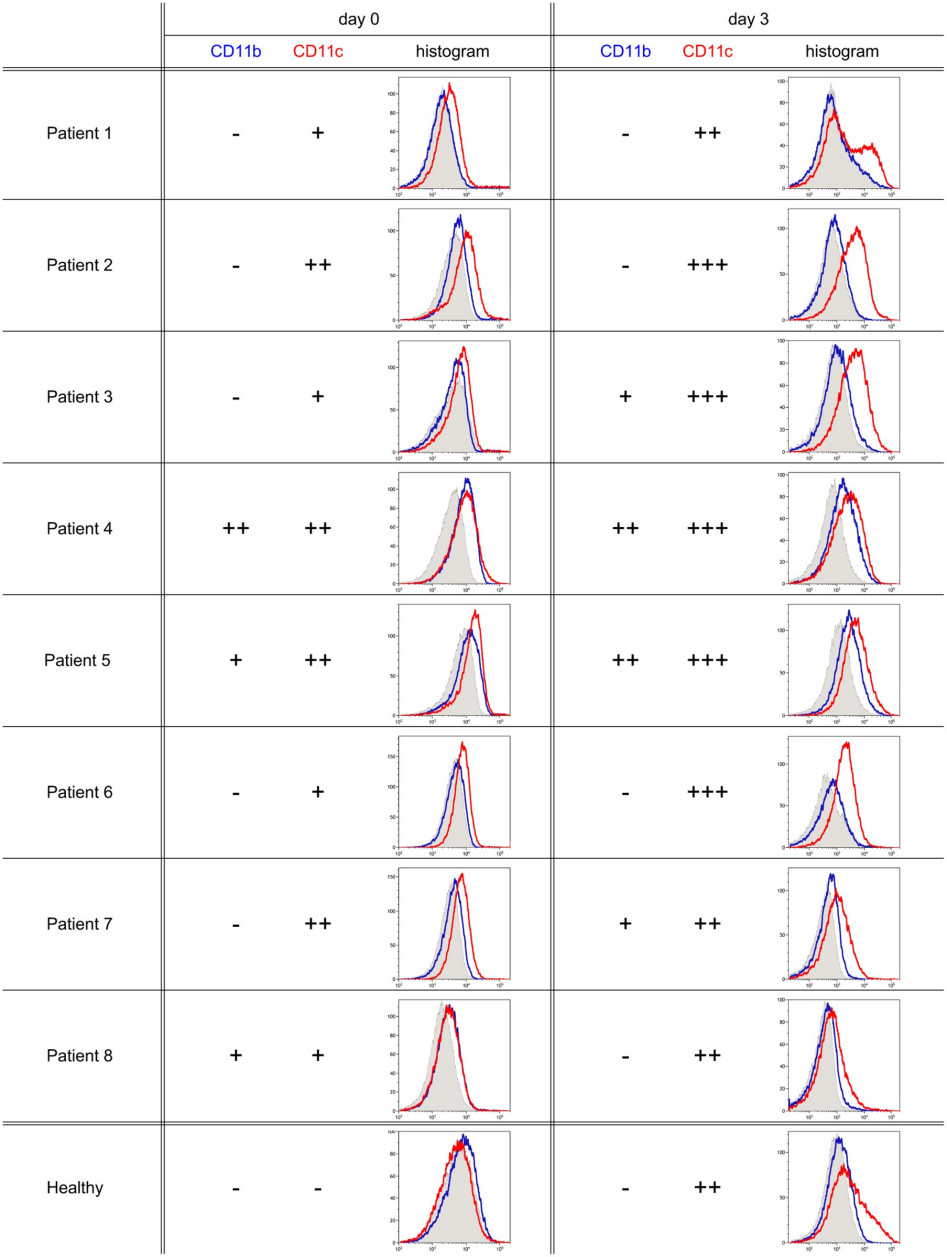
The expression of CD11b and CD11c on B cells of CLL patients directly after isolation and on the 3^rd^ day of culture. CR3 (blue) and CR4 (red) expression was measured by flow cytometry on B cells of eight CLL patients directly after isolation and on the 3^rd^ day of cell culture. As controls, B cells of healthy donors were investigated (in that case one representative histogram is shown of four independent experiments with similar results). On the histograms, the abscissa refers to the measured fluorescence intensity, while the ordinate refers to cell count. Expression was ranked positive if relative mean fluorescence intensity (RMFI) was higher than 150%. (RMFI = MFI of CD11b (clone ICRF44, blue) or CD11c (clone BU15, red) /MFI of isotype control (clone MOPC-21, grey). Positivity was ranked by the number of + symbol as follows: RMFI <150% ‘-‘, 150–200% ‘+’, 200–500% ‘++’, >500% ‘+++’).

B cells of CLL patients are known to express varying levels of CR3 and CR4 [4, 13–19], however, the function of these receptors on CLL cells and their contribution to the pathogenesis of the disease is unexplained. We selected eight CLL patients, whose B cells express at least one of these β_2_-integrins. Blood derived B cells were cultured in the presence of IL-2 and anti-IgG/A/M F(ab’)_2_ antibody, to ensure survival of the malignant cells. CD11b and CD11c expression was measured by flow cytometry directly after isolation and after 3-day culture. We found, that although unstimulated CLL B cells already express CD11c, in most of the cases the expression of this β_2_-integrin can be further stimulated by BCR activation, similarly to that found on B cells of healthy donors [11] (Fig 2). While all of the examined patients’ B cells expressed CD11c at each time point, the expression of CD11b varied by patients and time. As control, the expression of CD11b and CD11c on B cells isolated from the blood of healthy donors is shown. Further characteristics of receptor expression and function on healthy B cells are demonstrated in our previous paper [11].

### 2.3. Expression of α5β1 and αIIbβ3 on B cells of CLL patients

Beside CR3 and CR4 other integrins are also able to bind fibrinogen, such as α5β1 (CD49e/CD29), αvβ3 (CD51/CD61), or αIIbβ3 (CD41/CD61) [23]. As we analysed the function of CR3 and CR4 using one of their natural ligands, fibrinogen, we found important to test whether the above listed integrins are also present on the B cells of the CLL patients. We detected CD41a and CD49e on the B cells of Patient 8, while none of these integrins were expressed by the unstimulated B cells of healthy donors (Table 1). Moreover, both CD41a and CD49e expression increased after 3 days of BCR stimulation in the case of CLL patients, resulting in CD41a^+^ CLL B cells in four (Patient 2, 3, 4, 5) and CD49e^+^ CLL-B cells in six (Patient 2, 3, 4, 6, 7, 8) of the studied cases. In contrast, only CD49e was expressed by BCR stimulated B cells of healthy donors. (In the case of healthy donors, expression levels were calculated as mean of 4 independent experiments. BJAB cells were also tested, but none of these integrins were detected).

**Table 1:** Expression of CD41a, CD51 and CD49e on freshly isolated CLL B cells and on the 3^rd^ day of culture.

	day 0			day 3		
	CD41a	CD51	CD49e	CD41a	CD51	CD49e
Patient 1	-	-	-	-	-	-
Patient 2	-	-	-	+	-	+++
Patient 3	-	-	-	+	-	+
Patient 4	-	-	-	++	-	++
Patient 5	-	-	-	+	-	-
Patient 6	-	-	-	-	-	++
Patient 7	-	-	-	-	-	+
Patient 8	+	-	+	-	-	+++
Healthy	-	-	-	-	-	+

Expression was measured by flow cytometry on freshly isolated B cells of CLL patients and after three days of culture. In the case of healthy donors, expression levels were calculated as mean of 4 independent experiments. Positivity was ranked by the number of + symbol as follows: RMFI <150% ‘-‘, 150–200% ‘+’, 200–500% ‘++’, >500% ‘+++’.

### 2.4. Both CR3 and CR4 mediate adhesion of CLL B cells

**Fig 3:**
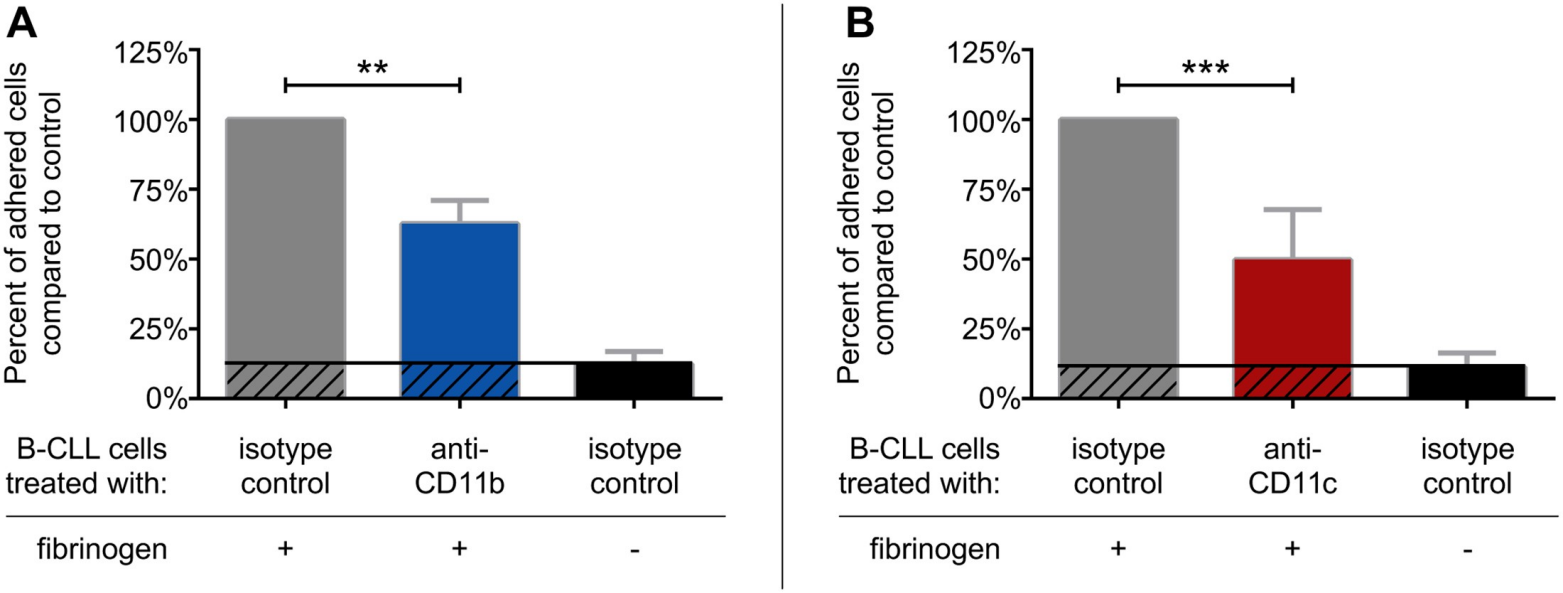
The effect of CD11b and CD11c specific antibodies on the adhesion of CLL B cells to fibrinogen coated and PLL-PEG blocked surface. The assay was carried out on CLL B cells on the 3^rd^ day of culture employing fibrinogen coated surfaces blocked by PLL-PEG or, as negative control, surfaces without fibrinogen Adherence was performed in the presence of (A) CD11b- or (B) CD11c- specific antibodies. (A) For CD11b results of four patients (Patient 3, 4, 5, 7), (B) for CD11c results of eight patients are summarized as mean +/- SD, normalized to control presented as 100%. One-way ANOVA with Tukey’s post-test was used to determine significant differences compared to control, * = p < 0.05; ** = p < 0.01; *** = p < 0.001.

Since CD11c was found to play a key role in the adhesion of activated B cells of healthy donors [11], as well as in the case of the Burkitt-like lymphoma cell line BJAB (Fig 1), we set out to investigate whether CR4 has a similar function on CLL B cells. Since in some cases CLL B cells were found to express CR3 as well, we also analysed the role of this β_2_-integrin in the adhesion to fibrinogen. We found, that blocking the function of either CR3 (blue) or CR4 (red) with specific antibodies significantly decreased the adherence of CLL B cells compared to the control samples (grey) (Fig 3). We used as negative control samples, where cells treated with isotype control antibody were let to adhere to a surface without fibrinogen coat (black). As seen in Fig 3, CR4 contributes to the adhesion of CLL B cells to fibrinogen, similarly to that observed in the case of BJAB cell line (Fig 1) and activated tonsillar B cells [11]. Moreover, we found that CR3, when expressed by CLL B cells, is also able to fulfil this function. We also tested how the simultaneous blocking of CD11b and CD11c affects the adhesion of the cells and found that the presence of anti-CD11b significantly augments the inhibitory effect of anti-CD11c. (S1 Fig). Interestingly, while both CD41a and CD49e were previously shown to mediate adhesion to fibrinogen on platelets [24] and on endothelial cells [25], the effect of CD41a and CD49e specific antibodies was not significant in the adhesion of CLL B cells (S2 Fig).

### 2.5. CR4 dominates over CR3 in the migration of CLL B cells towards SDF-1

**Fig 4:**
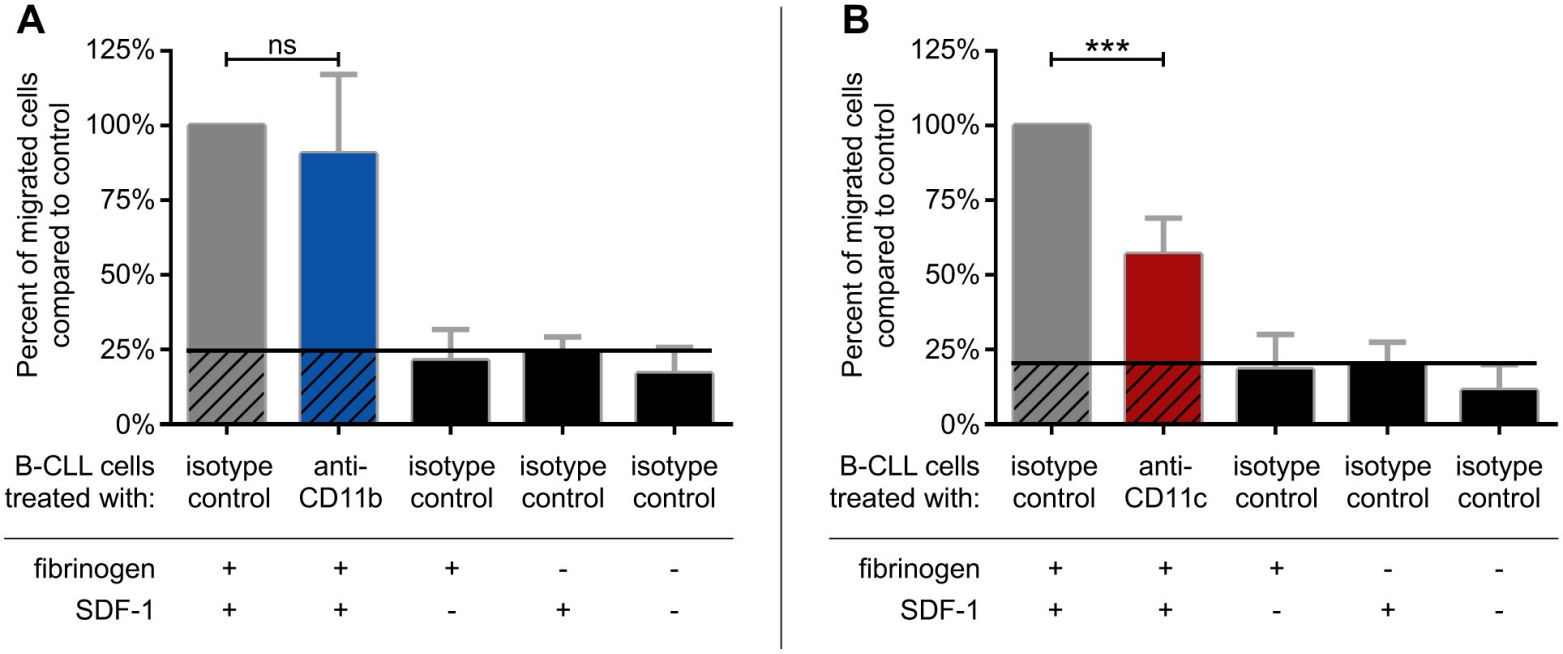
Migration of CLL B cells through fibrinogen coated and PLL-PEG blocked membrane towards SDF-1 can be blocked by CD11c specific antibody. (A) CD11b- or (B) CD11c- specific antibody was present throughout the assay, and the number of transmigrated cells were counted by flow cytometry. The assay was carried out on CLL B cells on the 3^rd^ day of culture. Results of four patients for CD11b (Patients 3, 4, 5, 7) and eight patients for CD11c are shown. Mean +/- SD, normalized to control is presented as 100%. One-way ANOVA with Tukey’s post-test was used to determine significant differences compared to control, * = p < 0.05; ** = p < 0.01, *** = p < 0.001.

Since the migration of leukemic cells plays an important role in the pathogenesis of the disease, we set out to analyse the involvement of CR3 and CR4 of CLL B cells in the migration through fibrinogen-coated and PLL-PEG blocked transwell membrane. We found that while the CD11b blocking antibody had no significant effect on migration (Fig 4A), inhibiting CR4 with a CD11c specific antibody resulted in a significant decrease in the number of migrated CLL-B cells (Fig 4B). This suggests that the capacity of the two β_2_-integrins is not identical, and CR4 dominates over CR3 in the migration of CLL-B cells. In the assays we used SDF-1 as chemoattractant, which is a key contributor to CLL pathomechanism, directing the malignant cells to the bone marrow, and providing survival signal to them [26, 27]. As negative control, we performed the assay in the absence of SDF-1 and/or using a transwell membrane which was not coated with fibrinogen, only blocked with PLL-PEG.

## 3. Discussion

Complement receptors CR3 and CR4 are widely expressed on myeloid cells, where CR3 has a dominant role in phagocytosis [2] while CR4 prevails over CR3 in adhesion to fibrinogen [3]. Though these complement receptors, which belong to the family of β_2_-integrins have earlier been thought to carry out overlapping functions, we suggest to consider them rather as “non-identical twins” [1], because of the functional segregation between them. Regarding their expression and role on B lymphocytes, we found recently that a proportion of B cells begin to express CD11c after BCR mediated activation, while CD11b could not be detected on B cells of healthy donors. The vast majority of CD11c^+^ human B lymphocytes belong to memory B cell subsets, and they use CR4 to mediate adhesion and migration on fibrinogen covered surfaces [11].

It is also known, that integrins are often overexpressed in malignant B cells, and the anatomical distribution of different B-cell lymphomas can be partially explained by the profile of the adhesion molecules expressed on their surface [28]. Amongst others, β_2_-integrins have been also detected on various B cell lymphomas, including diffuse large B-cell lymphoma, mantle cell lymphoma, Hodgkin’s lymphoma, CLL or Burkitt’s lymphoma [28]. While CR3 and CR4 are reported to appear also on B cells of patients suffering from Hodgkin’s lymphoma [12], hairy cell leukaemia [13] or CLL [4, 13–19], the role of these complement receptors on malignant B cells is still in question. Since CR4 was found to be involved in the adhesion and migration of activated B cells of healthy donors [11], we assumed that it retains its function on malignant B cells and may even serve as an active driver of the disease.

To answer this question, first we studied BJAB, the EBV-negative Burkitt lymphoma cell line, which expresses CD11c (Fig 1A). We found that CR4 maintains its function in adhesion to fibrinogen, proving its role also on this model of malignant B cells (Fig 1B). While cell lines can serve mainly as model, the results obtained provided important information regarding the function of CD11c and encouraged us to perform further experiment using the B cells of CLL patients.

Out of the mentioned CR3 and CR4 bearing B cell malignancies, CLL is the most common leukaemia in the western world. This disease is characterized by the monoclonal expansion of dysfunctional CD5-, CD19- and CD23-positive B cells in the blood, secondary lymphoid tissues and bone marrow, where they crowd out healthy blood cells [21]. BCR-dependent activation is a key factor in the pathogenesis of CLL, however, BCR-mediated responses are heterogeneous depending on numerous factors and vary from case to case [29]. One of these factors is the IGVH mutational status of the CLL cells, which divides the disease to mutated (M-CLL) and unmutated (U-CLL) cases. The outcome of antigen engagement in U-CLL differs from that in M-CLL [30], with U-CLL tending to have a lower affinity for antigen than affinity-matured M-CLL, which is related with the generally more aggressive behavior of unmutated cases [29]. However, the difference is not absolute, and antigen-responding B cells are apparently present in both U-CLL and M-CLL [30], presumably leading to proliferation and/or survival [29, 31].

Regarding CR3 and CR4 expression, varying percentage of patients were found to carry CD11c^+^ B cells, namely 21% [16], 26% [14], 27% [15], 40% [17], 49% [13] or even 89% [19]. The prevalence of CD11b varied between 20% [15] and 66% [16]. The presence of either CR3 or CR4 was found to be an unfavourable prognostic factor in B-CLL as the expression of both β_2_-integrins correlates with the pattern of bone marrow infiltration [14]. However, the exact mechanism of how these receptors contribute to the pathomechanism of CLL has not been revealed so far. Since CLL B cells display a homogeneous memory phenotype [22], we assumed, that CR4 may exert a similar function as on activated memory B cells of healthy donors [11]. Based on our earlier findings we focused on the adhesion and migration of CLL B cells.‬

In the present study, all the analysed patients’ B cells were positive for CR4, while only few patients carried CR3. Since we performed the functional studies using fibrinogen as ligand, and no systematic studies were done so far on the appearance of other fibrinogen-binding integrins (α5β1, αvβ3, and αIIbβ3), we tested their expression on CLL B cells, and as control, on normal B cells. Unstimulated B cells of healthy donors did not express any of these molecules, but after 3 days of BCR stimulation they began to express CD49e, which is in line with the findings of Ballard et al., who demonstrated VLA-5 expression on tonsillar and peripheral blood B lymphocytes stimulated by Staphylococcus aureus Cowan (SAC) [32]. In the case of CLL B cells we detected CD41a and CD49e on the freshly isolated B cells of Patient 8 (Table 1). Moreover, both CD41a and CD49e expression increased further after three days of the BCR stimulus.

Analyzing the contribution of CD41a and CD49e as well as of CR3 and CR4 to the adhesion to fibrinogen of malignant B cells we found, that both CR3 and CR4 were involved (Fig 3), while neither CD41a nor CD49e contributed significantly to this function of CLL B cells (S2 Fig). Importantly, CR3 and CR4 together were responsible for approximately 70% of fibrinogen-dependent adhesion in the case of the studied patients (S1 Fig), suggesting that other receptors also participate in this process. One of the candidates is ICAM-1 (CD54), which is known to interact with fibrinogen and is expressed at high levels in CLL cells [19, 33].

The stromal cell-derived factor-1 (SDF-1, also known as CXCL-12) has at least two major effects on CLL B cells; it causes migration towards stromal cells and provides survival signals as well [26]. The proliferating compartment of CLL exists in the bone marrow and lymph nodes, where the stromal microenvironment provides anti-apoptotic and pro-survival signals [27]. Moreover, increasing evidence suggests that the stromal microenvironment contributes to resistance to a wide variety of treatments. It has been observed that although therapies are often effective at killing CLL cells in the blood, residual cells causing disease remain in the bone marrow and lymph nodes [27], where the tumor microenvironment has been shown to promote chemoresistance [34]. For this reason, we analyzed the participation of CR3 and CR4 in the migration of CLL B cells towards SDF-1. We found that CR4 strongly contributes to the SDF-1 dependent migration of CLL B cells (Fig 4B) in contrast to CR3 (Fig 4A). Knowing the essential role of the stromal microenvironment in the pathomechanism of CLL, the involvement of CR4 to the migration towards SDF-1 vindicates the association of CD11c expression with bone marrow infiltration.

In conclusion, we revealed a mechanism, how CR3 and CR4 might contribute to the progression of chronic lymphocytic leukemia. Overcoming adhesion-mediated resistance is important in developing new therapies, as proven in the case of the integrin VLA-4. Natalizumab, the blocking anti-VLA-4 antibody, has been demonstrated to decreases B lymphocyte adherence to stroma and thereby partially control stromal protection toward rituximab and cytotoxic drugs [35]. We suggest that a similar effect can be expected from anti-CR4 and maybe even from anti-CR3 antibodies, which may even complete the partial effect of natalizumab and other treatments.

## 4. Materials and methods

### 4.1. Patients

Blood samples of patients diagnosed with chronic lymphocytic leukaemia were obtained from the Department of Internal Medicine and Oncology of Semmelweis University with their clinical data summarized in Table 2. None of these patients required therapy at the time of the study, they were monitored using a watch and wait strategy. The study was conducted according to the guidelines of the Declaration of Helsinki, and approved by the Hungarian Medical Research Council Scientific and Research Committee (ETT TUKEB, permission number: 21655-1/2016/ EKU). As controls, peripheral blood B lymphocytes were isolated from buffy coat obtained from healthy donors provided by the Hungarian National Blood Transfusion Service. Written informed consent was provided for the use of blood samples according to the Helsinki Declaration.

**Table 2:** Characteristics and clinical data of B-CLL patients at the time of the study.

	age (years)	sex	Rai stage	IGHV status
Patient 1	72	female	I	mutated
Patient 2	73	male	I	NA
Patient 3	65	female	I	mutated
Patient 4	67	male	I	mutated
Patient 5	47	female	I	mutated
Patient 6	71	female	I-II	mutated
Patient 7	66	female	II	NA
Patient 8	71	male	I	unmutated

### 4.2. Isolation of B cells

Peripheral blood mononuclear cells (PBMCs) were isolated by Ficoll-Hypaque (GE Healthcare, Chicago, IL, USA) density gradient centrifugation from patients’ EDTA-treated venous blood. Patients’ B cells were purified by negative selection using the Miltenyi B-CLL Cell Isolation Kit (Miltenyi Biotec, Bergisch Gladbach, Germany) achieving >97% purity, verified by CD19 expression. B cells of healthy donors were isolated from PBMC using the Pan B Cell Isolation Kit (Miltenyi Biotech). In some experiments BJAB, an African EBV-negative Burkitt-like lymphoma cell line obtained from American Type Culture Collection (ATCC; U.S.A.) was used.

### 4.3. Culture conditions

Chronic lymphocytic leukemic B cells were cultured in RPMI-1640 medium (Sigma-Aldrich, St. Louis, MO, USA) containing 10% FCS (Thermo Scientific, Rockford, IL, USA) and 50 μg/ml gentamycin at 37°C and 5% CO_2_, in the presence of 50 ng/ml IL-2 (ImmunoTools GmbH, Friesoythe, Germany) and 5 μg/ml goat anti-human IgG/A/M F(ab’)_2_ antibody (Jackson ImmunoResearch, Cambridgeshire, UK). Functional studies were carried out on the 3^rd^ day of the culture.

### 4.4. Flow cytometry

We carried out flow cytometry analyses directly after cell isolation and on the 3^rd^ day of cell culture. To characterize CLL B cells the following antibodies were used: anti-CD11b (clone ICRF44, IgG1, Biolegend, San Diego, CA, USA), anti-CD11c (clone BU15, IgG1, ImmunoTools GmbH, Friesoythe, Germany), anti-CD41a (clone HIP8, IgG1, Invitrogen, Thermo Fisher Scientific, Waltham, MA, USA), anti-CD51/CD61 (clone 23C6, IgG1, Invitrogen) anti-CD49e (clone SAM1, IgG2b, Invitrogen) with Alexa Fluor 647-conjugated goat anti-mouse antibodies (Thermo Fisher Scientific Inc.). As isotype control mouse IgG1 (clone MOPC-21, Biolegend) and mouse IgG2b (clone MPC-11, Biolegend) were used. To rule out dead cells from the analyses we used propidium iodid (Thermo Fisher Scien-tific Inc.) staining. Measurement was performed on a CytoFLEX cytometer (Beckman Coulter Life Sciences, Indianapolis, IN) using the CyteExpert software. Data were analysed using the CytExpert and Kaluza softwares.

### 4.5. Adhesion assay

Adherence of CLL B cells was assessed on the 3^rd^ day of the culture. We also measured the adhesive capacity of CD11c positive BJAB cells. Adhesion assay was carried out as described in our previous paper [11]. Namely, before and during the assay cells were incubated with Fc-receptor blocking reagent (Miltenyi Biotec) to avoid Fc-receptor mediated binding of the integrin-specific antibodies. For blocking the function of integrins, cells were treated for 30 minutes at 4 °C with 10 μg/ml of the following antibodies: anti-CD11b (clone ICRF44, IgG1, Biolegend), anti-CD11c (clone BU15, IgG1, ImmunoTools), anti-CD41a (clone HIP8, IgG1, Invitrogen), and anti-CD49e (clone SAM1, IgG2b, Invitrogen). All of the used integrin specific antibodies were previously shown to block the function of the target molecule [10, 11, 24, 36]. As control, isotype matched control antibodies were used (mouse IgG1, clone MOPC-21, Biolegend and mouse IgG2b, clone MPC-11, Biolegend). To ensure that integrins recycled from the cytoplasm are also blocked, the antibodies were not washed out for the assay. Ninety-six-well CELLview cell culture dish with glass bottom (Greiner Bio-One, Kremsmünster, Austria) was coated with 10 μg/ml fibrinogen (Merck, Budapest, Hungary) for 1 h at 37°C. After washing with PBS, free surfaces were blocked with 250 μg/ml synthetic copolymer PLL-PEG (ethylene glycol) (PLL-*g*-PEG, SuSoS AG, Dübendorf, Switzerland) for 1 h at 37 °C. As negative control, we measured the number of adhered cells to PLL-PEG blocked surfaces in the absence of fibrinogen coat. After blocking the cells with antibodies as mentioned above, they were allowed to adhere to the fibrinogen-coated and/or PLL-PEG blocked surfaces for 1 h at 37 °C and 5% CO_2_ in 100 μl of medium. After fixing with 2% paraformaldehyde (Sigma-Aldrich) for 10 minutes unbound cells were washed away with PBS and the adherent cells were stained with Draq5 (Biolegend) and phalloidin-Alexa488 (Molecular Probes, Thermo Fisher Scientific, Waltham, MA, USA) containing 0.1% Triton X-100 (Reanal, Budapest, Hungary). Images were taken by an Olympus IX81 laser scanning confocal microscope using the FluoView 500 software. Eight representative fields were scanned in two wells for each treatment and nuclei were counted using ImageJ software.

### 4.6. Migration assay

The measurement of migration was performed on CLL B cells on the 3^rd^ day of the culture. Migration assay was carried out as described in our previous paper [11]. Namely, before and during the assay cells were incubated with Fc-receptor blocking reagent (Miltenyi Biotec) to avoid Fc-receptor mediated binding of the specific antibodies. For blocking the function of integrins, cells were treated for 30 minutes at 4 °C with 10 μg/ml of anti-CD11b (clone ICRF44, Biolegend), anti-CD11c (clone BU15, ImmunoTools), or isotype matched control antibodies (mouse IgG1, clone MOPC-21, Biolegend). To ensure that integrins recycled from the cytoplasm are also blocked, the antibodies were not washed out for the assay. The migration assay has been performed using 24 well Transwell plates (polycarbonate membrane with 5.0 μm pore, Corning, NY, USA) towards 100 ng/ml SDF-1α (Thermo Fisher Scientific). Transwell membranes were coated with 100 μg/ml fibrinogen in PBS overnight at 37 °C and masked with 250 μg/ml PLL-PEG for 1 h at 37 °C. Antibody-treated cells were added to the upper chamber of the transwell assay in 100 μl RPMI-1640 medium containing 10% FCS and 50 μg/ml gentamycin, while the lower chamber of the assay contained 100 ng/ml SDF-1α (Thermo Fisher Scientific) diluted in 600 μl of the same medium. As negative control, we measured the number of migrated cells through PLL-PEG masked transwell membranes in the absence of fibrinogen coat and/or the chemoattractant SDF-1. After the cells were allowed to migrate for 4 h at 37 °C and 5% CO_2_, 25 mM EDTA was added to the lower chamber and the upper chamber was removed. Transmigrated cells were collected from the lower chamber, and cell number was counted immediately using a CytoFLEX cytometer (Beckman Coulter Life Sciences).

### 4.7. Statistics

For the functional analyses, we compared each treatment to the appropriate control sample, presented on the graphs as 100%. Statistical tests were performed with GraphPad Prism 6 software, with p<0.05 considered significant.

## Supporting information

S1 Fig The combined effect of CD11b and CD11c specific antibodies on the adhesion of CLL B cells to fibrinogen coated and PLL-PEG blocked surface. BCR activated CLL B cells were let to adhere to surfaces coated with fibrinogen and blocked by PLL-PEG or to surfaces without fibrinogen as negative control. Adherence was performed in the presence of CD11b- or CD11c- specific antibodies or both. Results of four patients (Patient 3, 4, 5, 7) are summarized as mean +/- SD, normalized to control presented as 100%. One-way ANOVA with Tukey’s post-test was used to determine significant differences, * = p < 0.05; ** = p < 0.01; *** = p < 0.001.
(TIF)

S2 Fig The effect of CD41a and CD49e specific antibodies on the adhesion of CLL B cells to fibrinogen coated and PLL-PEG blocked surface. Cells were let to adhere to surfaces coated with fibrinogen and blocked by PLL-PEG or to surfaces without fibrinogen as negative control. Adherence was performed in the presence of (a) CD41a or (b) CD49e- specific antibodies. For CD41a results of four patients (Patient 2, 3, 4, 5), for CD49e results of six patients (Patients 2, 3, 4, 6, 7, 8) are summarized as mean +/- SD, normalized to control presented as 100%. One-way ANOVA with Tukey’s post-test was used to determine significant differences compared to control.
(TIF)
