# Supplementary figures and images for: BCR activated CLL B cells use both CR3 (CD11b/CD18) and CR4 (CD11c/CD18) for adhesion while CR4 has a dominant role in migration towards SDF-1
Source: PLoS One. 2021 Jul 20;16(7):e0254853. doi: 10.1371/journal.pone.0254853 (PMC8291664; doi:10.1371/journal.pone.0254853)

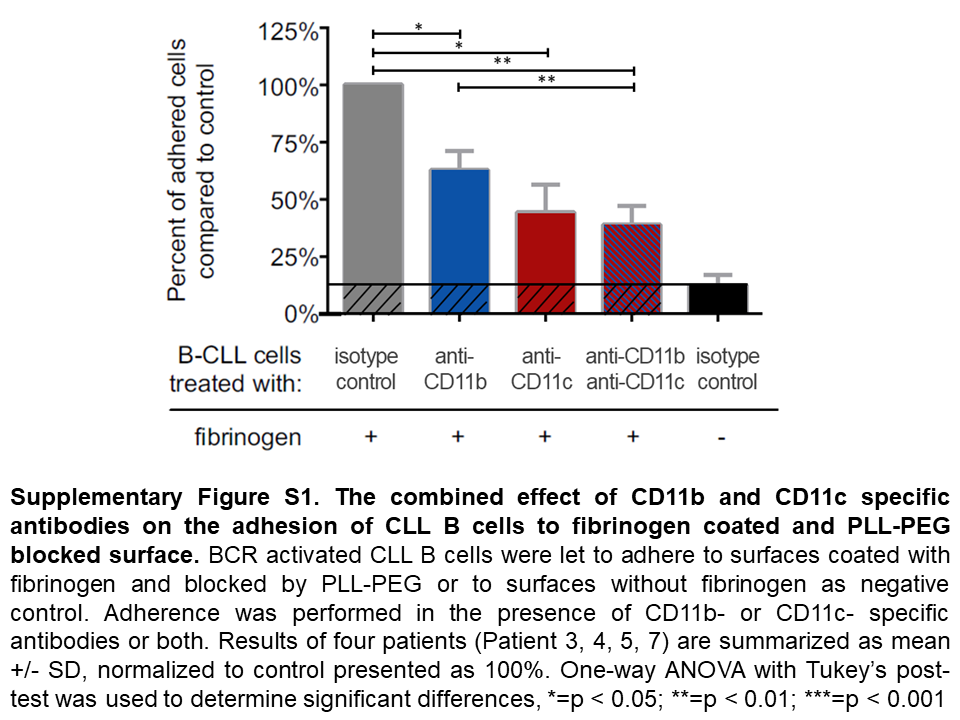

Supplement: S1 Fig — BCR activated CLL B cells were let to adhere to surfaces coated with fibrinogen and blocked by PLL-PEG or to surfaces without fibrinogen as negative control. Adherence was performed in the presence of CD11b- or CD11c- specific antibodies or both. Results of four patients (Patient 3, 4, 5, 7) are summarized as mean +/- SD, normalized to control presented as 100%. One-way ANOVA with Tukey’s post-test was used to determine significant differences, * = p < 0.05; ** = p < 0.01; *** = p < 0.001. (TIF) [file pone.0254853.s001.tif]

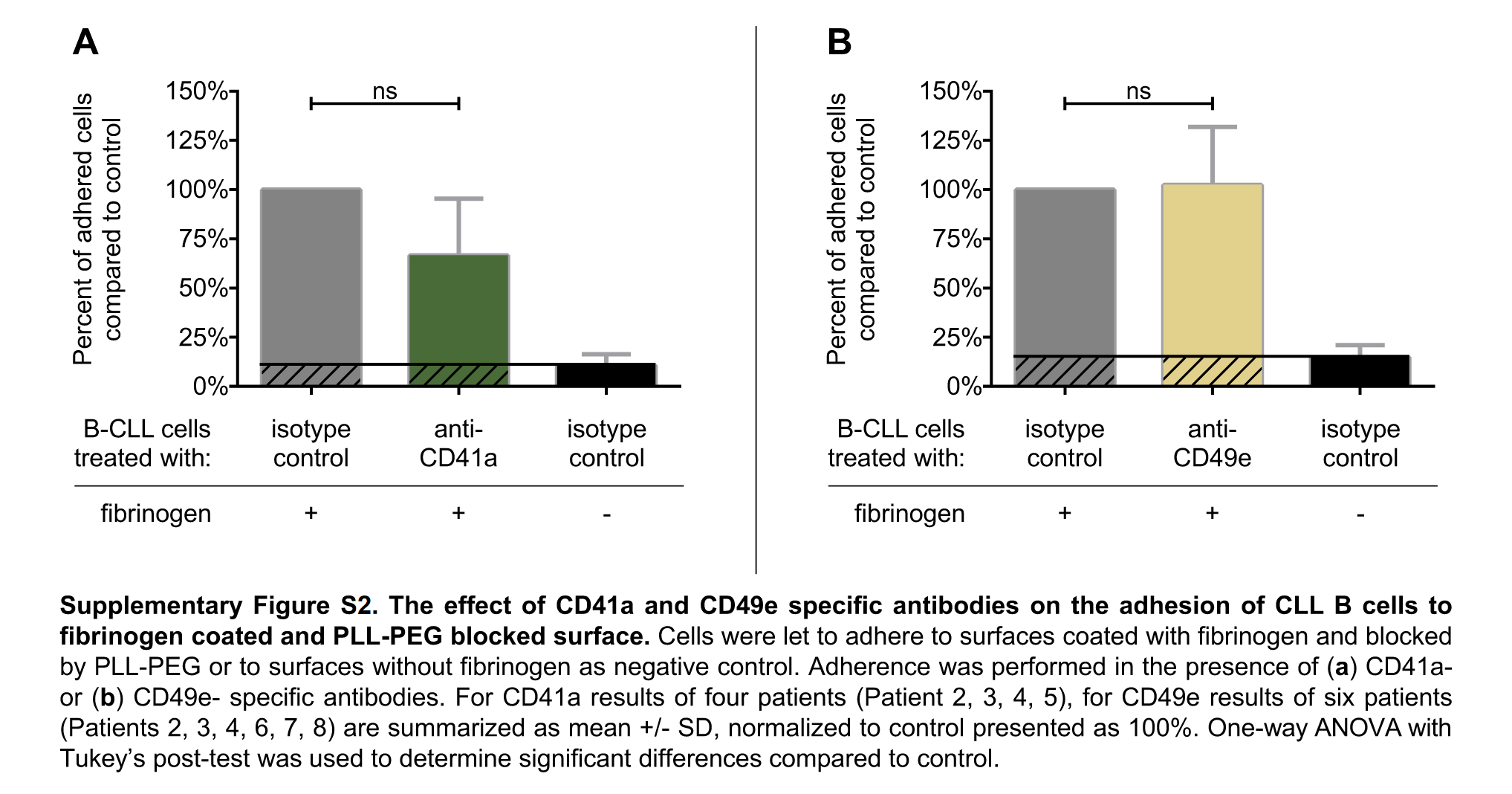

Supplement: S2 Fig — Cells were let to adhere to surfaces coated with fibrinogen and blocked by PLL-PEG or to surfaces without fibrinogen as negative control. Adherence was performed in the presence of (a) CD41a or (b) CD49e- specific antibodies. For CD41a results of four patients (Patient 2, 3, 4, 5), for CD49e results of six patients (Patients 2, 3, 4, 6, 7, 8) are summarized as mean +/- SD, normalized to control presented as 100%. One-way ANOVA with Tukey’s post-test was used to determine significant differences compared to control. (TIF) [file pone.0254853.s002.tif]
